# Neutrophil extracellular traps: New players in cancer research

**DOI:** 10.3389/fimmu.2022.937565

**Published:** 2022-08-19

**Authors:** Junjie Zhao, Jiaqi Jin

**Affiliations:** ^1^ Department of General Surgery, Changsha Hospital Affiliated to Hunan Normal University/The Fourth Hospital of Changsha, Changsha, China; ^2^ Department of Neurosurgery, The Second Affiliated Hospital of Harbin Medical University, Harbin, China

**Keywords:** neutrophil extracellular trap, tumor-associated neutrophils, tumor microenvironment, metastatic, therapy resistance

## Abstract

NETs are chromatin-derived webs extruded from neutrophils as a result of either infection or sterile stimulation using chemicals, cytokines, or microbes. In addition to the classical role that NETs play in innate immunity against infection and injuries, NETs have been implicated extensively in cancer progression, metastatic dissemination, and therapy resistance. The purpose of this review is to describe recent investigations into NETs and the roles they play in tumor biology and to explore their potential as therapeutic targets in cancer treatment.

## Introduction

An inflammatory response to tumors is a contributing factor to tumor initiation and progression, allowing cancer cells to escape detection by the immune system. Cancer progression depends on the interaction between tumor-infiltrating immune cells and tumor-derived factors in the tumor microenvironment (TME). Initially, infiltrating and resident immune cells in the TME contribute to tumor growth, metastasis, and responses to immunotherapy. According to conventional wisdom, macrophages are central players in the TME and are activated to respond in a wide variety way to local and circulating stimuli. The multiple roles played by neutrophils in circulating and infiltrating tumors have become increasingly clear in recent years.

Neutrophils represent 50–70% of all leukocytes in the body. As cells in the innate immune system, neutrophils play a prominent role in fighting against bacterial pathogenic infection and have been considered essential players in remodeling the TME *via* neutrophil extracellular trap (NET) generation or NETosis. NETs are extracellular DNA-integrated chromatin decorated with granular proteins, such as neutrophil-derived neutrophil elastase (NE) and myeloperoxidase (MPO) (1). In addition to the roles they play in host defense, NETs have been widely implicated in noninfectious diseases, such as autoimmune diseases (2–4), rheumatic diseases (5, 6), thrombosis (7–9), diabetes (10), and atherosclerosis (11–13). Increasing evidence indicates that NETs play a critical role in the TME, including in tumor progression, metastasis and therapy resistance (14–28). The effects of NET-related components seem to be intertwined and complicated.

In this review, we illustrate the mechanism and potential role played by NETs within the TME, highlighting their potential as cancer therapy targets.

### Tumor-associated neutrophils

Among circulating leukocytes in humans, neutrophils are the most abundant cell type. Neutrophils are often the first cells to reach sites of tissue injury or infection. Increasing evidence indicates that inflammation is closely linked to cancer development, and biomarkers of inflammatory reactions have been found to be useful as prognostics in several types of cancer. A highly heterogeneous TME affects tumor malignancy and treatment responses. Inflammatory cells and cytokines create an inflammatory TME, promoting tumor cell proliferation, survival, immune evasion, and migration (29, 30). The various innate immune cells in the TME include tumor-associated macrophages (TAMs), polymorphonuclear neutrophils (PMNs, also known as TANs) and myeloid-derived suppressor cells (MDSCs) (31). In the past two decades, the role played by TAMs in tumor biology has become relatively well established (32–35), but the role played by TANs in these processes is still largely unknown (36). Neutrophils are becoming increasingly recognized as important and contributors to cancer progression, not merely as bystanders in the TME.

Similar to macrophages, TANs undergo polarization state switching, which seems to be determined by the TME. The concept of an N1 and N2 neutrophil phenotype was first introduced by Fridlender in 2009 (37). Previous studies have demonstrated that G-CSF or transforming growth factor-β (TGF-β) are critical for the transformation of neutrophils toward the N2 phenotype (37, 38). Despite neutrophil polarization being reported in some diseases, no clinically relevant biomarker has been identified to distinguish N1 and N2 neutrophils in tumors, in contrast to reports describing M1 and M2 macrophage biomarkers.

## NETosis

In 2004, Brinkmann and colleagues demonstrated that activated neutrophils secrete NETs, which are web-like structures made of decondensed chromatin and various granular proteins (39). Initially, NETosis was thought to be the manifestation of a novel type of cell death that differs from necrosis and apoptosis. NETosis is triggered by a wide range of factors, including pathogens (40), activated platelets, and phorbol myristate acetate (PMA) (41). Peptidyl arginine-deiminase 4 (PAD4) changes the chromatin charge in neutrophils, leading to chromatin recondensation. In the presence of various stimuli, cytoplasmic granules and the nuclear membrane dissolve, and nuclear lobes are lost. Granule enzymes migrate to the nucleus, where they promote chromatin condensation and histone hypercitrullination (42, 43). To date, two forms of NETosis have been established. In one form, the lytic suicide mechanism, the rupture of cytoplasmic membranes is needed, whereas in the other form, vital NETosis, cytoplasmic membrane rupture is not required (44–47).

### Lytic NET formation

It was first described that NETs formed followed by cell death or release of lytic NETs.

Lytic NETosis can be caused by several factors, including PMA, autoantibodies, and cholesterol crystals. The activation of NADPH oxidase produces ROS, which activate PAD4 and decondense chromatin. Further chromatin unraveling occurs as a result of neutrophil elastase (NE) and myeloperoxidase (MPO) translocation. Chromosomes are decorated with granular and cytosolic proteins when they enter the cytosol. The neutrophil dies when its plasma membrane has been disrupted, releasing NETs.

### Nonlytic NET formation

The time required for lytic NET formation has been reported to occur primarily at 3 to 4 hours. In contrast, neutrophils can also release NETs in a very rapid (5-60 min) and cell death-independent manner. When S. aureus and E. coli enter the body, their complement receptors, as well as TLR2 and TLR4 receptors, are activated within minutes. Activating PAD4 causes chromatin decondensation, possibly without oxidizing agents. Suicidal NETosis occurs when NE translocates into the nucleus, causing chromatin to unfold. Neutrophils retain their ability to carry out further functions, such as phagocytosis, because of expulsion of protein-decorated chromatin through vesicles.

The functional complexity of neutrophils is closely related to the diversity of their components (42, 43). Histones and DNA are the major components of NETs in various studies (39, 48, 49). Furthermore, many neutrophil granule components, such as NE, MPO, cathepsin G, and proteinase 3 (PR3), are essential components of NETs (42, 43, 50). Recent research has suggested that NETs may participate in various disorders, including diabetes (10), autoimmune diseases (3, 51, 52), atherosclerosis (11–13), thrombosis (7, 53), and cancer (14–18, 26, 54, 55). In the past five years, the study of NETs has provided new insights into the TME.

## The role of NETS in cancer biology

**Figure 1:**
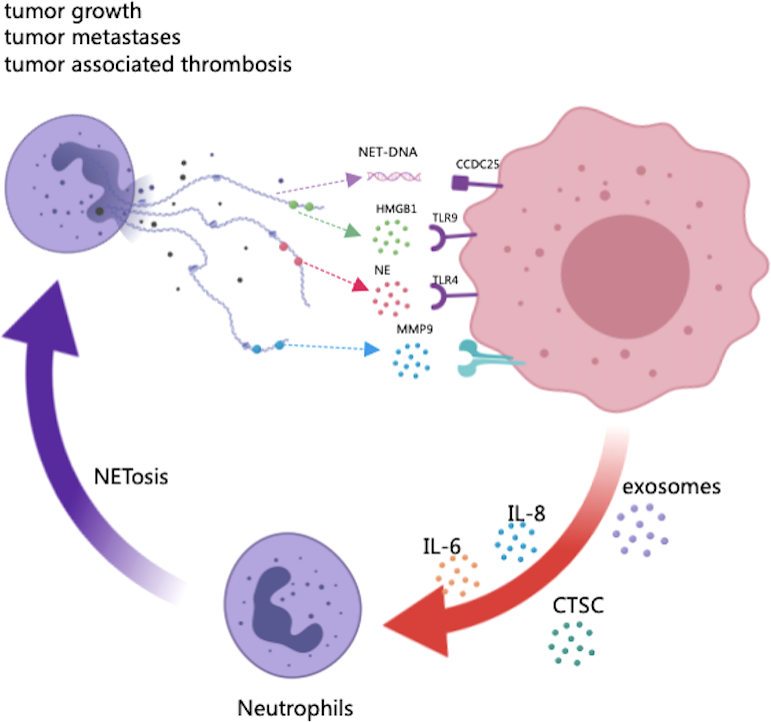
The potential role of NETs in tumor progression and metastasis. As scaffolds, NETs capture cancer cells and provide a microenvironment in which protumor genic proteins can be delivered to cancer cells. As part of NETs, HMGB1 is released, activating TLR9-dependent pathways in cancer cells. The NE released by NETs triggers the TLR-4 receptor on cancer cells, resulting in the upregulation of PGC-1, increased mitochondrial biogenesis, and accelerated growth. The transmembrane protein CCDC25 on cancer cells senses extracellular DNA and activates the ILK-parvin pathway to enhance cell motility. In turn, certain factors secreted by many primary tumors have been shown to promote NET formation, such as cytokines (HIF-1, IL-8, IL-6), exosomes and proteases (CTSCs).

Increasing recognition has been made of the importance of NETs in the TME. Some explorations into the potential biological role played by NETs in tumors, including tumor growth, metastasis and therapy resistance, have been initiated. Recent studies describing the relationships between NETs and cancer are shown in **Table 1** and **Figure 1**. The following paragraphs will describe the role of NETs in cancer biology in further detail (**Table 2**).

**Table 1:** Tumor-associated neutrophils (TANs) in tumors.

Mechanism	References
**T/NK-cell suppression**	
Arginase 1	(56)
PR3	(57)
MPO/Hydrogen peroxide	(58, 59)
NETs	(14, 16–18)
ROS	(60)
**Angiogenesis/Metastasis**	
BV8	(61, 62)
VEGF	(63)
MMP9	(64)
NAMPT/STAT3	(65)
S100A4	(66)
NETs	(19, 23, 25–27)
Elastase	(67)
Oncostatin M	(68, 69)
**Tumor Cytotoxicity**	
ROS	(70)
Granzyme B	(71)
MET signaling	(72)
Ferropotosis	(73)
Elastase	(74)

**Table 2:** NETs in cancer.

Cancer types	References
Pancreatic cancer	(18, 75–85)
Liver cancer	(19, 24, 27, 86–91)
Colon cancer	(17, 92–99)
Gastric cancer	(100–106)
Small intestinal cancer	(107)
Gallbladder cancer	(108)
Thyroid cancer	(109)
Breast cancer	(23, 25, 26, 110–124)
Glioma	(125, 126)
Lung cancer (127–137)	
Head and neck cancer (138, 139)	
Esophageal cancer (106)	
Acute Promyelocytic Leukemia (140, 141)	
Diffuse Large B-cell Lymphoma (22).	
Myeloproliferative neoplasms (112, 142)	
Multiple myeloma (143)	
Melanoma (144)	
Bladder cancer (55, 145)	
Oral squamous cell carcinoma (146–150)	
Endometrial cancer (151)	
Ovarian cancer (20, 152, 153)	
Cervical cancer (154)	

### NETs: Novel biomarkers in cancer patients

In the first decade after NET discovery, research on NETs was primarily focused on infectious diseases and circulatory system-related diseases because neutrophils are the most abundant circulating immune cells and exhibit antibacterial activity. NETs have been reported in human solid tumors, with the first observation reported in 2013. This early NET study focused on a small number of Ewing sarcoma samples and suggested that patients with intertumoral NETs had a significantly shorter life expectancy than patients without intertumoral NETs (155). In recent decades, NETs have been considered to be putative biomarkers in patients with various types of cancers. Evidence has suggested that plasma NETs are potential biomarkers for predicting early-stage cancer and tumor metastasis in 73 patients with head and neck cancer (HNC) (138). According to this HCN study, NETs were less common in advanced cancer stages (T3–4, N3), which correlates with an increase in granulocyte colony-stimulating factor (G-CSF) levels. In another study focused on small cohorts of cancer patients, citrullinated histone H3 (CitH3), the gold standard for NET formation identification, was found to be a prognostic blood signature for patients with advanced cancer, as suggested by Melanie Demers et al. (156). Based on their study, high levels of CitH3 powerfully heralds poor clinical results for cancer patients. NETs have also been observed in the peripheral blood, lung tissue, and sputum of lung cancer patients (127). The levels of NETs generated by neutrophils stimulated with IL-8 and LPS from colorectal cancer patients were obviously higher than those in healthy controls and were associated with a poor clinical outcome (157).

### NETs in tumor growth

A TME is often a hypoxic complex environment replete with cytokines and growth factors. The necrosis of tumor cells caused by the hypoxic microenvironment often leads to the release of danger-associated molecular patterns (DAMPs), which cause inflammation. Hamza O et al. demonstrated that NETs induced increased mitochondrial function in tumor cells, supplying energy for accelerated tumor growth. NET formation in the hypoxic TME is caused by chemokines and high mobility group box 1 (HMGB1) levels, which have been found to be high in conditioned medium prepared with hypoxic cancer cells. In PAD4-knockout (KO) mice, the progression of tumor growth and hepatic metastases were both extremely slow compared to those in wild-type mice. The expression of a number of mitophagy-associated proteins, including DRP-1, MFN-2, PINK1 and Parkin, was increased in cancer cells exposed to NETs. TLR-4 was activated *via* NE, and mitochondrial biogenesis and lymphocyte proliferation were found in the tumors of PAD4-KO mice (28).

In another study, PAD4-deficient mice did not show reduced tumor growth, but the administration of exogenous G-CSF induced intertumoral NET formation in the wild-type (WT) host. These results suggested that the tumor or TME primes neutrophils for NET formation and leads to the accumulation of intertumoral NETs and a growth advantage for tumors (158).

NET levels were high in patients with advanced diffuse large B-cell lymphoma (DLBCL) and were closely related to low survival rates in a previous study. Tumor progression was promoted by the activation of Toll-like receptor 9 (TLR9), which is in a downstream pathway activated by lymphoma-derived IL-8. In preclinical models, blocking the IL-8-CXCR2 axis or TLR9 activation delayed tumor progression (22).

Several cancers are associated with chronic inflammation and infection, as these conditions promote cancer progression. NETs have been proven to be key threads in the transformation from an inflammatory state to cancerogenesis. A study revealed that NETs were induced by sustained inflammation that awakened dormant cancer cells (128). Smoking or inhalation of lipopolysaccharide (LPS) caused chronic lung inflammation that resulted in NET formation, inhibiting lung tumor growth both *in vitro* and *in vivo*. Mechanistically, through the MAPK/ERK/MLCK/YAP signaling pathway, NET-mediated proteolytic remodeling of laminin caused an epitope to activate dormant cancer cells, promoting their proliferation (128).

### NETs in tumor metastasis

Neutrophils can facilitate angiogenesis in primary tumors by releasing MMP9, S100A8/9, and BV8 to activate VEGF (61, 62, 64, 159, 160). Neutrophil elastase (NE) and MMP9 can promote tumor cell proliferation by releasing growth factors and degrading laminin (67, 128). Furthermore, inflammatory stimuli (IL-1β and TNF-α) can stimulate neutrophil MET expression and HGF binding, leading to NO production and tumor cell death (72). From the perspective of the primary tumor site, neutrophils can promote metastasis by promoting cancer cell escape into blood vessels (161). In the circulation, neutrophils facilitate the progression of circulating tumor cells through their cell cycle (162). It has been shown that neutrophils can direct disseminated cancer cells to specific sites and promote vascular leakiness for easy extravasation (161, 163, 164). Additionally, neutrophils can release protein-coated nucleic acids, called neutrophil extracellular traps (NETs), that catch circulating cancer cells and stimulate cancer growth.

Due to their special structures, NETs have received considerable attention because of their potential role in tumor metastasis (19, 20, 25–27). For example, in orthotopic ovarian cancer models, researchers found that neutrophil influx into the omentum was a precursor to metastatic progression (20). The inflammatory factors derived from ovarian tumors stimulated neutrophils to secrete NETs. Moreover, NETs were observed in the omentum of ovarian tumor-bearing mice before metastasis and in patients with early-stage carcinoma. These NETs bound ovarian cancer cells and may have promoted tumor metastasis, according to the study. Genetic and pharmacological blockade of PAD4 expression notably decreased omental metastasis.

NETs have also been observed in metastatic lung lesions, but they exhibit the highest seroprevalence rate in triple-negative tumors (23). These researchers found that breast cancer cells initiated NETosis to promote metastasis. In mice orthotopically transplanted with murine breast cancer cells, CXCL1 mediated neutrophil recruitment to the TME. Compared to 4T07 (nonmetastatic) tumors, primary 4T1 (metastatic) tumors exhibited a higher number of neutrophils, and blocking CXCL1 expression in 4T1 cells diminished neutrophil infiltration into tumors. Additionally, metastatic cancer cells induced NETosis through the release of G-CSF at sites of NET dissemination, and inhibition of G-CSF release prevented the formation of NETs by 4T1 cells.

In recent years, various reports have discussed the importance of NETs in tumor metastasis, but the definite mechanism for this phenomenon remains unclear. The expression of a5b1, avb3, and avb5 integrins allowed cancer cells to adhere to NETs derived from neutrophil-like cells *in vitro*. Cancer cells were inhibited from adhering to NETs by the cyclic RGD peptide to a degree comparable to the effect of DNase (21).

In studying the interaction between NETs and metastases, a recent report provided new insight into a NET-DNA receptor on cancer cells (26). According to this novel finding, liver metastases in breast and colon cancer patients were associated with high NET levels in patient serum, and the risk for liver metastases in patients with breast cancer in the early stages was higher than the risk for patients with colon cancer. In cancer cells, the NET-DNA receptor CCDC25 senses extracellular DNA and promotes cell mobility through the ILK–β-parvin pathway. In the clinic, Yang et al. found that CCDC25 expression on primary cancer cells predicted poor outcomes. Based on their original studies, they explained how NETs interact with cancer cells and identified CCDC25 as a DNA sensor involved in the interaction between NETs and cancer cells.

A recent report revealed a novel mechanism by which tumor cells controlled NETosis in metastatic niches. They found that cathepsin C, a tumor-secreted protease, promoted breast-to-lung metastasis by priming the formation of NETs (25). Neutrophil membrane-bound proteinase 3 (PR3) is activated by CTSC to accelerate the progression of interleukin-1β (IL-1β) and nuclear factor κB activation. This activation resulted in increased IL-6 and CCL3 production, leading to NETosis neutralization.

### NETs in tumor-associated thrombosis

The occurrence of thrombosis is common in most cancers and is closely linked to cancer patient mortality (165–167). Venous thromboembolism (VTE) has been reported to develop in 4% to 20% of children, and arterial thrombosis has been reported in 2% to 5% of children (168–170). Many factors contribute to cancer-associated thrombosis, but the underlying mechanisms remain unclear. Many different biomarkers have been analyzed with the aim of identifying cancer patients at high risk for VTE. Recently, an interest in identifying procoagulant and prothrombotic factors has been generated (53, 171). Furthermore, the first report on NETs in cancer was focused on the potential role played by NETs in cancer-associated thrombosis (129). These early studies showed that malignant neutrophils were likely to form NETs in a chronic myelogenous leukemia mouse model. Cancers cause an expansion in peritoneal NETs through a systemic effect on the host, as shown by the increased likelihood of NET formation in mammary and lung carcinoma models. One study showed that the expression of JAK2V617F was associated with NET formation and thrombosis in patients with Philadelphia chromosome-negative myeloproliferative neoplasms (MPNs) (112).

### Other cell types of ETs

In addition to neutrophils, other immune cells, such as eosinophils (172), dendritic cells (173), monocytes/macrophages (75, 174), mast cells (175), basophils (176), and lymphocytes (177, 178), may also generate extracellular traps (ETs), indicating possible multiple origins of extracellular DNA in cancer. However, because the different cell types of ETs share the biomarker citH3, there are no accurate methods that can differentiate NETs and other cell types of ETs in tumor microenvironments. Moreover, due to a lack of studies on other cell types of ETs, the mechanism of ET formation in different cell types is still largely understood. It will be necessary to conduct future research to understand the definite mechanism of ET formation and explore the potential clinical translational value of ETs in cancer treatment.

## NETs in cancer therapy resistance

**Figure 2:**
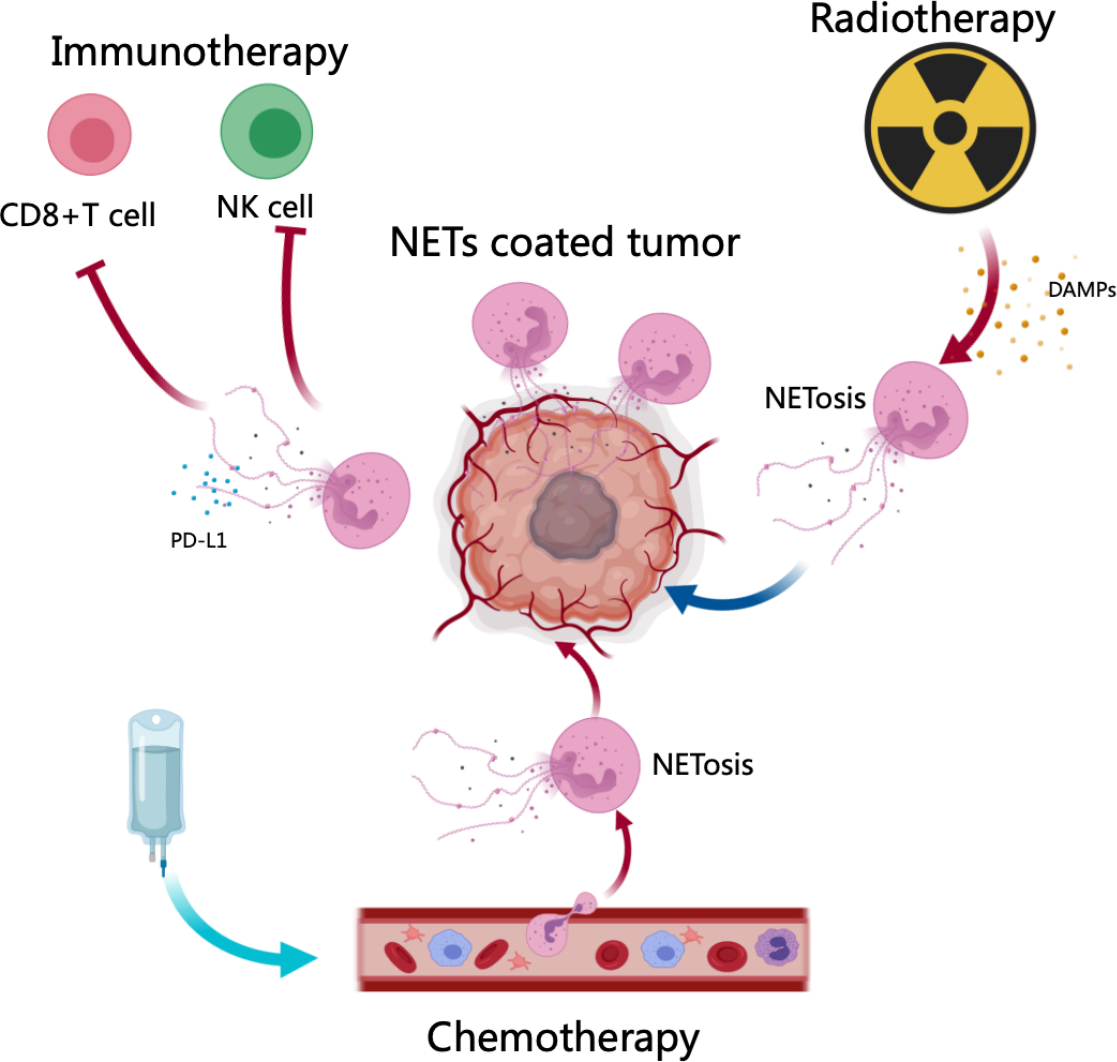
NETs in cancer therapy resistance. After chemotherapy and radiotherapy, DAMPs (HMGB1) or other signals from cancer cells induce NET generation, contributing to therapy resistance. Neutrophils in tumor microenvironments expelled NETs, which protected tumor cells from cytotoxicity through CD8+ T cells and NK In addition, NETs decorated with PD-L1 neutralized the function of CD8+ T cells, resulting in checkpoint blockade in immunotherapy.

As a result of recent basic and clinical research implicating NETs in resistance to cancer therapy, the status of NETs as a research topic is rapidly changing (14–18, 54, 55, 179). As chemotherapy, radiation therapy, and immunotherapy are essential for cancer treatment, new strategies must be developed to mitigate resistance. NETs are becoming increasingly relevant in cancer therapy resistance, and we review the recent studies that support this relevance (**Figure 2**).

### Neutrophils in cancer therapy resistance

There is increasing evidence that TANs play an important role in the TME, which is thought to play a central role in resistance to cancer therapy (9, 33–38). As a result of their production of cytokines and chemokines, neutrophils are known to potentiate the survival mechanisms of cancer cells and therefore inhibit the response to therapies (6, 7, 36, 39–45). In recent years, it has been proposed that neutropenia is more than just an indication of sufficient therapeutic dosing but also of resistance mechanisms dependent on TAN (21, 39, 46, 49). It is well known that neutrophils play an important role in cancer treatment resistance, leading to an interest in NETosis as a potential mechanism (127, 138, 156, 157).

### NETs in chemotherapy resistance

A limited number of studies have examined the clinical association between the level of NET formation and response to chemotherapy, with preliminary *in vitro* and *in vivo* data indicating that NETosis is a mechanism of chemoresistance. For instance, multiple myeloma (MM) cells were chemoresistant after treatment with doxorubicin (179). In the presence of anthracycline drugs such as doxorubicin, NETs are internalized by neoplastic cells, detoxifying the microenvironment. In animal models, degradation of NETs by DNase restored chemosensitivity, supporting the idea that NETs contribute to chemoresistance. Although, to our knowledge, few studies have been reported describing NETs in chemoresistance, extracellular traps (ETs) in acute promyelocytic leukemia (APL) cells have been observed after treatment with all-trans retinoic acid (ATRA) and arsenic trioxide (ATO) (140, 180–183). These findings are noteworthy since they suggest that NETs or ETs may be potential therapeutic targets for improving chemotherapy responses.

### NETs in radiotherapy resistance

Although radiation therapy is a common treatment for cancer, a significant proportion of patients develop resistance, making it difficult to control tumors locally. In addition to immunological changes in the TME postradiotherapy, neutrophils have been studied for their novel roles in radiotherapy resistance (184, 185). In addition, recent research has indicated that neutrophils play a functional role in radiotherapy resistance through the formation of NETs (54, 55). Studies have also shown that neutrophils play a pivotal role in radiotherapy resistance by forming NETs. After tumor irradiation (IR), NETs form in tumor microenvironments, contributing to radiotherapy resistance. NE inhibitors (NEi) and DNase 1 were effective in inhibiting NETosis and NET degradation, respectively, and they thus increased the effectiveness of radiation therapy, suggesting that these inhibitors may be used to enhance radiation therapy. In addition, researchers have found that NETs were more likely to form in tumors of patients with a poor response to radiation treatment, which correlated with poor outcomes. A combination of NET-based clinical exploration may be a novel and promising treatment for radiotherapy resistance.

It is well described that the enzyme ectonucleotide pyrophosphatase/phosphodiesterase 1 (ENPP1) helps regulate soft tissue mineralization as well as skeletal mineralization (186). ENPP1 is well recognized for its roles in purinergic signaling, a form of signaling that is inextricably linked with cancer incidence (187, 188). Breast cancer patients with local recurrence or failure (LRF) after surgery and IR have a dismal prognosis. Notably, Enpp1 caused LRF in a newly developed and refined animal model established in a recent study (54). High expression of Enpp1 in circulating tumor cells (CTCs) results in relapse, requiring PMN-MDSC and NET infiltration within tumors. Enpp1 inhibition or genetic and pharmacological NET blockade might prolong relapse-free survival (54). Enpp1-derived adenosinergic metabolites increased Haptoglobin (Hp) expression, causing myeloid invasion and NETosis. In relapsed human breast cancer tumors, ENPP1 and NET levels were notably elevated. A combination of NET inhibitors may attenuate radiotherapeutic resistance in clinical trials.

### NETs in immunotherapy resistance

Cancer immunotherapy has attracted considerable attention in recent years. The composition of the TME profoundly affects the success of immunotherapy. The molecular mechanisms underlying immune checkpoint blockade resistance are poorly understood and have been the subjects of intense scrutiny in recent years. In several recent studies, NETs have been examined as novel mediators in some tumors that resist checkpoint inhibition (14–18). Using a PDAC model, Zhang et al. demonstrated that IL17 triggered the formation of NETs within tumors, which can lead to the exhaustion of cytotoxic CD8+ T cells in tumors. In addition, IL17 blockade enhanced tumor cell sensitivity to immune checkpoint blockade. Moreover, abrogating NETs through genetic and pharmacological treatment can lead to the same immunotherapy resistance phenotype, indicating that NETs participate in immunotherapy resistance.

Agonists of CXCR1 and CXCR2 have been demonstrated to cause the development of NETs in 4T1-bearing mice, protecting cells from immune cytotoxicity and negatively affecting the efficacy of checkpoint inhibitors (16). NETs coat tumor cells, preventing them from contacting CD8+ T cells and natural killer cells. NETs are necessary for this protection against cytotoxicity because DNase 1 treatment restored effector-target contact and subsequently cancer cell death. Intravital microscopy experiments were performed with lung carcinoma model mice, and the findings confirmed this mechanism of NET protection *in vivo*.

A novel mechanism in which T-cell exhaustion is regulated by NET formation in liver ischemia/reperfusion (I/R) in a cancer metastasis model was proposed in a recent report (14). In this study, NETs in the tumor microenvironment inhibited the response of T cells by inducing T-cell metabolic and functional exhaustion, thereby enhancing tumor growth. The treatment of mice with DNase inhibited NET formation *in vivo*, resulting in attenuated tumor growth, a reduced NETosis rate and higher levels of functioning T cells. Immunosuppressive effects of PD-L1 on T-cell exhaustion in the presence of NETs have been reported. Experiments with clinical samples from patients with colorectal liver metastases validated these PD-L1 effects.

NETs have been explored in various types of cancers in recent years. Whether NETs participate in immunotherapy resistance in these different types of cancers has not been fully investigated. In addition, although NETs have been shown to promote checkpoint resistance in previous studies, the underlying mechanism of this process remains unclear. Does a DNA sensor similar to CCDC25 bind NETs on the surface of T cells? If this sensor is present on T cells, could it be a new marker for predicting the effectiveness of immunotherapy?

Considering the present findings, further investigation into NET-targeting therapeutics in combination with immunotherapy is warranted for patients who would otherwise have poor responses to immunotherapy (**1**, **2**).

## Conclusions

Crosstalk between NET formation and the TME indicates the ways NETs contribute to cancer progression and metastasis. NETs can promote cancer growth, metastasis and treatment resistance to chemotherapy, checkpoint inhibitors and radiotherapy. However, despite the increasing interest in NETs in potential cancer therapies, more studies are required to explore the possibility of pharmacologically interfering with NET formation.

## Author contributions

Conceptualization: JZ. Writing original draft preparation: JJ. Writing editing: JJ. Figures: JZ and JJ. All authors contributed to the article and approved the submitted version.

## Funding

This work was supported by the Natural Science Foundation of Changsha City (kq2202026).

## Acknowledgments

We thank Ge Mang for technical assistance in creating the figures in this manuscript.

## Conflict of interest

The authors declare that the research was conducted in the absence of any commercial or financial relationships that could be construed as a potential conflict of interest.

## Publisher’s note

All claims expressed in this article are solely those of the authors and do not necessarily represent those of their affiliated organizations, or those of the publisher, the editors and the reviewers. Any product that may be evaluated in this article, or claim that may be made by its manufacturer, is not guaranteed or endorsed by the publisher.

## Abbreviations

APL, acute promyelocytic leukemia; ATO, arsenic trioxide; ATRA, all trans retinoic acid; CitH3, citrullinated histone H3; CTCs, circulating tumor cells (CTCs); DAMPs, danger-associated molecular patterns; DLBLC, diffuse large B-cell lymphoma; HMGB1, high mobility group box 1; HNC, head and neck cancer; IL-1β, interleukin-1β;LRF, local recurrence or failure; MDSC: myeloid-derived suppressor cells; MM, multiple myeloma; MPN, myeloproliferative neoplasms; MPO, myeloperoxidase; NE, neutrophil elastase; NETs, neutrophil extracellular traps; PAD4, Peptidyl arginine-deiminase 4; PMA, phorbol myristate acetate; PMN, polymorphonuclear neutrophil; PR3, proteinase 3; TAMs, tumor-associated macrophages; TAN, Tumor-associated neutrophil; TME, tumor microenvironment; TNF, tumor necrosis factor; VTE, Venous thromboembolism; WT, wild type.
